# Detection of paroxysmal atrial fibrillation in 994 patients with a cerebrovascular event by intermittent 21-day ECG-monitoring and 7-day continuous Holter-recording

**DOI:** 10.48101/ujms.v127.8318

**Published:** 2022-05-05

**Authors:** Johanna Pennlert, Mårten Rosenqvist, Milos Kesek

**Affiliations:** a Department of Public Health and Clinical Medicine, Umeå University, Umeå, Sweden; b Danderyd University Hospital and Karolinska Institutet, Stockholm, Sweden; c Department of Public Health and Clinical Medicine and Heart Center, Umeå University, Umeå, Sweden

**Keywords:** Arrhythmia, ischemic stroke, TIA, continuous ECG-monitoring, intermittent ECG-recordings, screening

## Abstract

**Background:**

The detection of paroxysmal atrial fibrillation (AF) is of importance in stroke care. The method used is continuous electrocardiogram (ECG)-monitoring or multiple short ECG-recordings during an extended period. Their relative efficiency is a matter of discussion. In a retrospective cohort study on 994 patients with an ischemic stroke or transient ischemic attack (TIA), we have compared continuous 7-day monitoring to intermittent recording 60 sec three times daily with a handheld device during 3 weeks. We related the result to subsequent occurrence of AF as detected in 12-lead ECG recordings.

**Methods:**

The patients were identified in the local database of cardiovascular investigations. Their clinical profile and vital status during the follow-up were obtained from the Swedish Stroke Register and the Swedish general population registry. For comparison, we used an age- and sex-matched population with no known cerebrovascular event and a population with a cerebrovascular event that was not screened.

**Results:**

AF was detected in 7.1% by continuous screening and in 5.1% by intermittent screening (*P* = 0.3). During follow-up of 32 months, AF in 12-lead ECG was found in 7.0%. In the subgroup with positive screening, 46.3% had AF compared with 6.7% in the subgroup with negative screening (*P* < 0.0001).

**Conclusions:**

The two screening approaches had a similar yield of arrhythmia, in spite of the group with intermittent monitoring having a more favorable clinical profile. A positive screening was highly predictive of AF in ECG during the follow-up.

## Introduction

The detection of silent paroxysmal atrial fibrillation (AF) is of importance in stroke care. The method of choice has been continuous electrocardiogram (ECG)-monitoring of successively longer duration (1, 2). More recently, multiple short ECG-recordings obtained intermittently have come in use (3–6). The latter method is easier to handle for patients with preserved functional level and covers a longer time period. The relative efficiency of these two strategies is a matter of discussion. The aim of this study was to compare the two screening methods in a retrospective, observational cohort study with follow-up at a single tertiary center.

## Materials and methods

Patients seeking Umeå University Hospital with cryptogenic stroke or transient ischemic attack (TIA) and without known AF are routinely referred to screening for paroxysmal AF. Two methods are used: continuous 7-day ECG-monitoring (LifeCard, Spacelabs Healthcare, WA, USA) and intermittent monitoring during 3 weeks with a handheld device capable of storing multiple ECG recordings of 60 sec duration (MyDiagnostick Medical, Maastricht, The Netherlands). In the latter, the patients are requested to make a recording three times daily and when symptomatic. The choice between the methods is primarily based on present equipment availability. The operator, however, considers the individual patient’s ability to handle the equipment. Subsequently, all the recordings are manually reviewed by experienced readers, and the results are summarized in a written statement and entered into the local database of cardiovascular investigations (PAF3, Medsharp, Stockholm, Sweden, www.medsharp.se). AF is defined as irregular supraventricular arrhythmia of at least 30 sec duration (7) with atrial activity of variable morphology or no visible atrial activity. Patients with a newly diagnosed AF are considered for oral anticoagulation treatment.

### Data sources

The local database PAF covers the cardiovascular investigations performed at Umeå University Hospital. The Swedish general population registry comprises data on all citizens’ vital status. The Swedish Stroke Register (Riksstroke, https://www.riksstroke.org) collects data from all hospitals admitting patients with cerebrovascular events, with an estimated nationwide coverage of 91% and 86% of all patients treated for stroke and TIA, respectively. The database MUSE-Västerbotten contains all the 12-lead ECG recordings obtained within the public health care of the Swedish county of Västerbotten (MUSE, GE Healthcare, Wauwatosa, WI, USA).

### Study population

We have retrospectively included all patients undergoing first-time AF-screening at Umeå University Hospital as workup after a cerebrovascular event (a stroke or a TIA) between April 2014 and April 2019. Procedures of both types were retrieved from the local database. All the procedures were initiated after a specific request for the screening of arrhythmia as embolic source after a cerebrovascular event. The reports were retrospectively classified with respect to occurrence of AF (author MK). The clinical profile was retrieved from Riksstroke. At this stage, only patients with ICD-10 diagnosis of TIA (G45) and ischemic stroke (I63) were included. The result of the screening was related to subsequent occurrence of clinical AF as detected in 12-lead ECG. In the ECG-database MUSE-Västerbotten, we identified the patients’ ECG recordings that were acquired after the initial request for AF-screening. The ECG recordings were classified with respect to the occurrence of AF or atrial flutter as detected by the automatic interpretation (12SL, GE Healthcare or the Glasgow algorithm, GRI, University of Glasgow, Glasgow, UK).

An administrative follow-up period was defined as the time between the initial request for the AF-screening and time of death or time of closure of the ECG-database (May, 2019). The patients’ vital status was determined from the general population registry. The first ECG with AF during follow-up was used as a substitute for the occurrence of clinical arrhythmia.

### Two comparison groups

Comparison group 1 contained patients from Umeå University Hospital and the Västerbotten county hospitals of Lycksele and Skellefteå, with a first TIA or ischemic stroke (with ICD-10 diagnoses G45 and I63 in Riksstroke) during 2014–2019 that were not screened for AF following the hospital stay. Their clinical profile was identified in Riksstroke. The date of the cerebrovascular event was used as the start of the follow-up period. Their ECG recordings were identified in the ECG-database MUSE-Västerbotten. The vital status during the follow-up was retrieved from the general population registry.

Comparison group 2 was an age- and sex-matched population with no known cerebrovascular events, which was constructed from the ECG-database MUSE-Västerbotten. We identified all subjects having a first-time ECG recorded between April 2014 and April 2019, being in sinus rhythm and not included in the study group. A random sample was chosen by 5× oversampling within strata that were matched to the study group with respect to sex and age in decades. Subjects with AF detected within 31 days and subjects dying within 31 days after the index ECG in sinus were excluded in order to remove subjects with AF secondary to an acute condition or perioperatively, subjects suffering perioperative death, and patients admitted for terminal illness. The date of the index-ECG was used as the start of the follow-up period. The subjects’ vital status during the follow-up period was identified, and the subsequent ECG recordings were checked for the occurrence of AF or atrial flutter.

This study complies with the Declaration of Helsinki and was approved by the Ethics Committee of the Medical Faculty at the Umeå University and the Swedish Ethical Review Authority (2018-156/31, 2019-05681).

### Statistics

Continuous parameters are given as median (Q1 and Q3) unless stated otherwise. Two-tailed Mann–Whitney test was used to test for differences between groups in continuous variables. Two-tailed Fisher exact test was used to test for differences in proportions. Differences in mortality were evaluated using the log rank test. The effect of the clinical parameters and their interactions on AF-detection by the two methods was studied in a multiple logistic regression model. A *P*-value < 0.05 was considered significant. R 4.0.3 (R Foundation for Statistical Computing, 2020, http://www.R-project.org) was used for statistical analyses.

## Results

**Figure 1:**
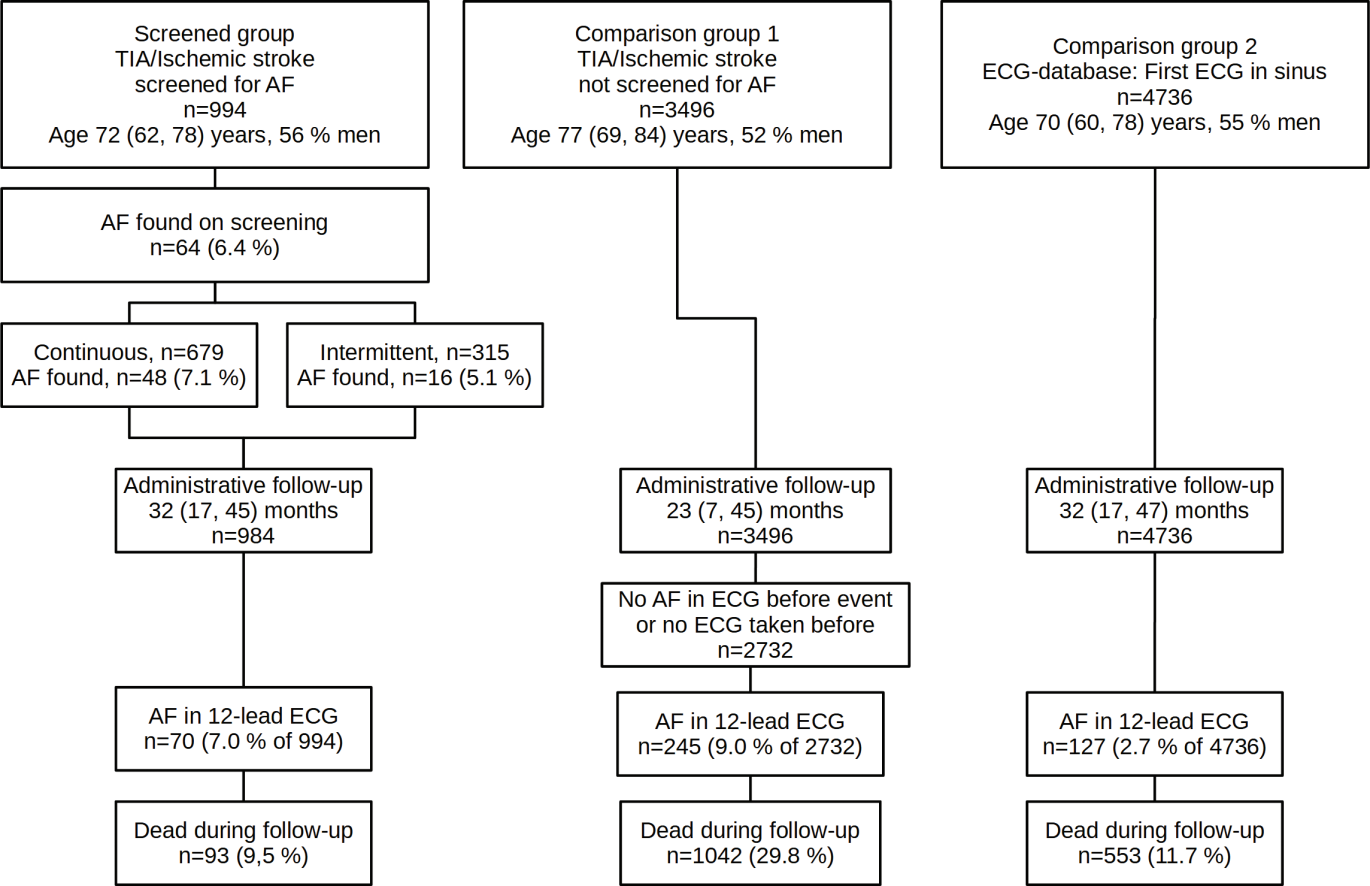
Study flow chart, screened and non-screened patients following stroke or TIA (study group and comparison group 1), and age- and sex-matched patients (comparison group 2) in sinus rhythm at baseline. Age is given as median (quartile 1 and quartile 3). Integer numbers are reported as n (percentage). TIA: transient ischemic attack; AF: atrial fibrillation; ECG: electrocardiogram.

The study included 994 patients and comprised 2,621 person-years of follow-up. The patient flow is summarized in Figure 1.

### Screening

Screening for AF with either method was performed in 994 patients (56% men), aged 72 (62, 78). The time-span from referral to actual screening was 27 (4, 67) days. AF was detected in 64/994 patients (6.4%). In 849 patients with TIA or ischemic stroke, the clinical profile could be obtained from Riksstroke (Table 1). Fewer patients with TIA screened positive for AF compared to patients with ischemic stroke (17/385, 4.4% vs. 41/464, 8.8%, *P* = 0.01, Fisher).

**Table 1:** Baseline data.

Parameter	Study group	Comparison group 1	Study vs. Comparison 1
Underwent screening for AF, *n* (%men)	994 (56)		
Retrieved from Riksstroke, *n* (%men)	849 (56)	3,496 (52)	
Age, years (for retrieved from Riksstroke)	72 (63, 79)	77 (69, 84)	*P* < 0.0001
Hypertension, *n* (%)	368 (57)	1,824 (65)	*P* = 0.0005
Diabetes, *n* (%)	102 (16)	548 (19)	*P* = 0.04
Drowsy or unconscious on admission, *n* (%)	23 (5)	312 (14)	*P* < 0.0001
Treated with anticoagulants* on admission, *n* (%)	8 (0.9)	387 (11)	*P* < 0.0001
In-hospital ECG-monitoring**, *n* (%)	211 (25)	825 (24)	*P* = 0.8
AF known during the medical care episode***, *n* (%)	27 (3)	903 (26)	*P* < 0.0001
Diagnosis TIA (ICD-10 diagnosis G45), *n* (%)	385 (45)	1,135 (32)	*P* < 0.0001
ADL help needed, *n* (%)	13 (2.8)	241 (10.8)	*P* < 0.0001
Follow-up duration, months (Q1, Q3)	31 (18, 45)	23 (7, 45)	*P* < 0.0001
Dead during follow-up, *n* (%)	87 (10)	1,042 (30)	*P* < 0.0001
Recurrent stroke during follow-up, *n* (%)	77 (9)	221 (6)	*P* = 0.006

AF: atrial fibrillation; ADL: activities of daily living; ECG: electrocardiogram; TIA: transient ischemic attack.

Note: The table shows the study group (patients with ischemic stroke or TIA, screened for AF) and the comparison group 1 (patients not screened for AF).

* Warfarin or non-vitamin K antagonist oral anticoagulant.

** Some patients underwent ECG-monitoring with telemetry during the hospital stay.

*** Some patients were registered as diagnosed with AF before or during the hospital stay in the comparison group 1 and, to a small degree, in the study group. In the latter, the screening may have been the mode of diagnosis.

### Continuous and intermittent screening

Continuous and intermittent monitoring were used in 679 and 315 patients, respectively (Table 2). AF was detected by continuous screening in 7.1% and by intermittent screening in 5.1% (*P* = 0.3, Fisher). A multiple logistic regression model was constructed with AF detection by screening as outcome parameter and chosen screening method, age, sex, diabetes, hypertension, and diagnosis (TIA vs. ischemic stroke) as predictors. In the final model, based on the screening method, age, diagnosis, and their interactions, only age remained as significant predictor (*P* = 0.01). The screening method and diagnosis were not significant (*P* = 0.14 and 0.11, respectively).

**Table 2:** Patients screened for AF with continuous versus intermittent screening methods.

Parameter	Continuous	Intermittent	Continuous vs. Intermittent
*n* (%men)	679 (53)	315 (61)	*P* = 0.03*
Age, years (Q1, Q3)	72 (62, 79)	71 (62, 78)	*P* = 0.4
Actual screening extent (Q1, Q3)	168 (168, 168) h	61 (56, 65) recordings	
Hypertension, *n* (%)	238 (58)	130 (7)	*P* = 0.9
Diabetes, *n* (%)	62 (15)	40 (18)	*P* = 0.4
Drowsy or unconscious on admission, *n* (%)	18 (6)	5 (4)	*P* = 0.6
Diagnosis TIA, *n* (%)	247 (43)	138 (51)	*P* = 0.02
Screening positive for AF, *n* (%)	48 (7.1)	16 (5.1)	*P* = 0.3
ADL help needed, *n* (%)	12 (3.6)	1 (0.8)	*P* = 0.12
Administrative follow-up, *n*	674	310	
Follow-up duration, months (Q1, Q3)	38 (21, 50)	22 (14, 32)	*P* < 0.0001
Dead during follow-up, *n* (%)	76 (11.3)	17 (5.5)	*P* = 0.003
ECG during follow-up positive for AF, *n* (%)	62 (9.1)	8 (2.5)	*P* = 0.0001
Recurrent stroke during follow-up, *n* (%available)	53 (9)	24 (9)	*P* = 1

TIA: transient ischemic attack; AF: atrial fibrillation; ADL: activities of daily living; ECG: electrocardiogram.

* For the sex.

### Follow-up

Survival data were available in 984 study patients (Figure 1). During the follow-up (median 32 months), 9.5% of the patients died. At least one ECG with AF during the follow-up was found in 7.0% of the study group. In the subgroup with positive screening for AF and an ECG recorded during follow-up, 25/54 (46.3%) had at least one ECG with AF compared with 45/669 (6.7%) in the subgroup with negative screening (*P* < 0.0001, Fisher). This corresponds to an estimated annual incidence of clinical AF of 17.6 and 2.6%, respectively. Among the 17 patients with TIA and positive screening, seven (41%) had AF in ECG during follow-up. In the 41 patients with ischemic stroke and positive screening, 17 (41%) had AF during follow-up.

### The comparison groups

The comparison group 1, retrieved from Riksstroke, consisted of 3,496 patients with a TIA or ischemic stroke (Table 1). During the follow-up (median 23 months), 30% of the patients died. In 9.0%, AF was identified in an ECG during the follow-up (Figure 1).

The comparison group 2, retrieved from the ECG-database, contained 4,736 subjects. During the follow-up (median 32 months), 11.7% died. At least one ECG with AF was found during the follow-up in 2.7%.

## Discussion

### Detection of AF by screening

In our study, paroxysmal AF was detected by screening in 6.4% of the patients. This is comparable to the 5.7% detected by a 7-day Holter recording by Jabaudon et al. (8) and the 6–7% detected by a 5-day Holter recording by Tuna et al. (9).

### Intermittent and continuous screening methods

The intermittent group had a more favorable clinical profile than the group with continuous monitoring (see Table 2). Possibly, an adverse clinical profile did bias the operators’ choice toward continuous monitoring. The two screening approaches, nevertheless, had a similar yield of arrhythmia. In a multiple logistic regression model, the screening method did not reach significance as predictor for AF detection on screening.

Poulsen et al. found similar yields of AF for continuous and intermittent screening after a cerebrovascular event (5). AF was detected in a fourth of their patients, in spite of slightly shorter continuous recording times and fewer intermittent recordings than in our study. Their population was 7 years older than in our material, presumably contributing to the markedly high prevalence of AF in their study. Fredriksson et al. compared 14 days continuous to 14 days intermittent recording in a mass screening setting. AF was found in 2 and 6% with the intermittent and continuous methods, respectively (3). The participants were 5 years older than the patients in our study, but their general population would have a lower prevalence of AF than our patients with cerebrovascular morbidity.

### AF during follow-up

We found AF in ECG during the follow-up in 7.0%, corresponding to an incidence of 26.4 per 1,000 person-years. This was somewhat higher than the incidence of 21.7 per 1,000 person-years in a study of AF during follow-up after a stroke (10). The two studies differed with respect to the definitions of included cerebrovascular events and the mode of diagnosing AF during follow-up. We could see that AF at screening was highly predictive of AF in ECG during the follow-up. The latter was found in 46.3 and 6.7% among patients with and without AF at screening, respectively, corresponding to an annual incidence of clinical AF of 17.6 and 2.6%, respectively. Similarly, in studies of patients with cardiac implantable electronic devices, the detection of subclinical AF is strongly related to a finding of clinical AF during follow-up (11). In the ASSERT study, the incidence of clinical AF was 6 and 1% in the groups with and without subclinical AF (12), respectively.

Screening for AF was positive in a smaller share of patients with TIA than in those with stroke. However, among those with positive screening for AF, the risk for AF during follow-up was similar in TIA and stroke. The relation between AF on screening and AF in subsequent ECG, thus, does not seem to vary with severity of the cerebrovascular event.

AF in ECG during follow-up was found in 9.0 and 2.7% of the comparison groups 1 and 2, respectively (Figure 1).

### Mortality during follow-up

The mortality during the follow-up was much lower in the study group than in the comparison group 1. The latter was in median 5 years older and had a smaller share of TIA and a greater share of impaired consciousness on admission (Table 1). This unfavorable clinical profile may have contributed to the difference. The mortality was also slightly lower in the study group than in the comparison group 2. Subjects with AF or suspected acute morbidity were not included in the latter. Nevertheless, indication for an ECG seemingly defined a mixed group with a considerable medium-term mortality that was unrelated to clinical AF.

### Recurrent stroke during follow-up

New stroke occurred in 9% of the patients in the study group compared with 6% in the comparison group 1 (Table 1). This difference may seem surprising, but it should be interpreted in the perspective of the very large mortality in the latter group.

When AF actually is detected in an individual patient with cryptogenic stroke, anticoagulation is strongly considered. The problem of screening by a method with limited duration is different. The diagnostic yield increases with the duration of monitoring (13), but no method with a limited time-span will exclude an intermittent arrhythmia.

The commonly used CHA_2_DS_2_VASC-score has not been validated in this setting and is probably not an entirely adequate measure. The continuous and the intermittent screenings will miss a proportion of patients with paroxysmal AF. It seems logical that intermittent screening should detect paroxysmal AF in a smaller proportion of subjects with a higher AF-burden compared with continuous recording. This has been shown in a modeling experiment on a large clinically non-specified dataset (14). Time-limited screening methods with a lower yield may still be adequate for detecting AF at a level of arrhythmia burden in a defined population where anticoagulation carries a benefit. Such a level will exist, since it is known that 1) anticoagulating patients with permanent AF reduces the risk of thromboembolic stroke (15), 2) anticoagulating all patients with cryptogenic stroke regardless of arrhythmia carries no benefit (16), 3) studies indicate that patients with paroxysmal AF have a smaller risk for stroke than patients with continuous arrhythmia, the relative risk being estimated to 0.7–0.8 (17, 18), 4) a greater arrhythmia burden increases the thromboembolic risk (19, 20), and 5) anticoagulation reduces the relative thromboembolic risk by 60–70% (21) but increases the risk of intracranial hemorrhage. AF is, however, a condition that progresses (22, 23), and it is a complex task to define a level at which undetected arrhythmias are less important.

In conclusion, screening with intermittent ECG-monitoring three times daily during 3 weeks has a yield of AF-detection comparable to the conventional 7-days continuous Holter recording. Patients suffering a cerebrovascular event have a higher subsequent occurrence of clinical AF than comparable controls. A finding of AF on screening of patients with cerebrovascular event is associated with a higher risk of subsequent clinical AF.

### Limitations

The retrospective nature of the material is a restriction. A large prospective randomized trial would be required for controlling the differences in the basic profiles of the groups and a possible bias in the evaluation of the ECG-recordings. Although the actual screening time and actual number of performed intermittent recordings are high, the quality of the individual recordings is varying. Recordings with AF in the ECG database will correspond to clinical AF during the follow-up. They are not a direct measure of prevalence of AF, due to the known large proportion of silent arrhythmia.
